# Modulation of Functional Connectivity Between Dopamine Neurons of the Rat Ventral Tegmental Area *in vitro*

**DOI:** 10.3389/fnint.2019.00020

**Published:** 2019-06-25

**Authors:** Luuk van der Velden, Martin A. Vinck, Taco R. Werkman, Wytse J. Wadman

**Affiliations:** ^1^Center for Neuroscience, University of Amsterdam, Amsterdam, Netherlands; ^2^Ernst Strüngmann Institute for Neuroscience in Cooperation With Max Planck Society, Frankfurt am Main, Germany

**Keywords:** dopamine, network, functional connectivity, glutamate, potassium, action potentials, multi-electrode array

## Abstract

Micro Electrode Arrays were used to simultaneously record spontaneous extracellular action potentials from 10 to 30 dopamine neurons in acute brain slices from the lateral Ventral Tegmental Area (VTA) of the rat. The spike train of an individual neuron was used to characterize the firing pattern: firing rate, firing irregularity and oscillation frequency. Functional connectivity between a pair of neurons was quantified by the Paired Phase Consistency (PPC), taking the oscillation frequency as reference. Under baseline conditions the PPC was significantly different from zero and 42 of the 386 pairs of VTA neurons showed significant coupling. Fifty percent of the recorded dopamine neurons were part of the coupled VTA network. Raising extracellular potassium from 3.5 to 5 mM increased the mean firing rate of the dopamine neurons by 45%. The same increase could be induced by bath application of 300 μm glutamate. High potassium reduced the PPC, but it did not change during the glutamate application. Our findings imply that manipulating excitability has distinct and specific consequences for functional connectivity in the VTA network that cannot be directly predicted from the changes in neuronal firing rates. Functional connectivity reflects the spatial organization and synchronization of the VTA output and thus represents a unique element of the message that is sent to the mesolimbic projection area. It adds a dimension to pharmacological manipulation of the VTA micro circuit that might help to understand the pharmacological (side) effects of e.g., anti-psychotic drugs.

## 1. Introduction

The ventral tegmental area (VTA) is a midbrain nucleus alongside the substantia nigra. The VTA plays a role in emotional processing, in the reward system and it is implicated in cognitive functions such as associative learning and memory (Lisman and Grace, 2005; Fields et al., 2007; Fujisawa and Buzsáki, 2011; Kim et al., 2012). The most abundant neuron type in the VTA is the dopamine neuron (Nair-Roberts et al., 2008) which projects to mesolimbic and mesocortical structures. Dopamine neurons form local synaptic connections with each other (Bayer and Pickel, 1990) but they are also interconnected with glutamatergic and GABAergic neurons (Omelchenko and Sesack, 2009). Many details of this local microcircuit are still under intense investigation. *In vivo* VTA dopamine neurons exhibit spontaneous activity and generate single action potentials (spikes) as well as bursts, with consequences for dopamine release (Gonon, 1988; Paladini and Roeper, 2014). Most remarkably they demonstrate a low quite regular firing rate (1–8 Hz) and this spontaneous activity even persists, at a somewhat lower frequency, in the *in vitro* brain slice which is devoid of external input (Grace and Onn, 1989; Bowery et al., 1994; Werkman et al., 2001; Bayer et al., 2007). The firing pattern can theoretically be explained by the composition of the ion conductances in the membrane wherein calcium currents and/or persistent sodium currents (Khaliq and Bean, 2010; Drion et al., 2011) play a prominent role. The VTA network, and in particularly the dopaminergic neurons, seem also sufficiently synchronized to generate a low frequency oscillation in the local field potential that could well be relevant for memory (Lisman and Grace, 2005; Fujisawa and Buzsáki, 2011) and noise correlations that support reward processing (Kim et al., 2012; Moghaddam et al., 2017). Model network studies show that the strength of the local functional connectivity is a major factor in generating such population oscillations (Traub et al., 1989). Anatomical and physiological studies have indicated considerable heterogeneity in cellular properties of DA neurons with consequences for their firing pattern and potentially linked to distinct output connectivity (Lammel et al., 2008). We have restricted our study to the mesolimbic projecting neurons in the lateral part of the VTA, where the classical slow firing, high DAT containing neurons are located (Björklund and Dunnett, 2007; Lammel et al., 2008). Most pharmacological studies in the VTA use the firing rate of individual dopaminergic neurons as their output parameter (Hand et al., 1987; Wang and French, 1993; Werkman et al., 2001) and indeed binding of various antipsychotic drugs to D_2_ receptors on these neurons leads to modulation in their firing rate (Pucak and Grace, 1994, 1996). In Substantia Nigra dual patch clamp recordings demonstrated that direct dopaminergic chemical transmission (as well as electrical) results in coupling (Vandecasteele et al., 2008). In previous work we have shown that the VTA neurons are functionally connected (van der Velden et al., 2017) and this connectivity is in part organized by the volume transmission of dopamine. If they are partially synchronized, drug manipulation at the receptor level might affect the organization of the local VTA network. This network effect of pharmacological manipulation can be determined by simultaneous recording from a sufficiently large sample of VTA dopamine neurons and analyzing their mutual relations. Micro Electrode Arrays (MEA) (Taketani and Baudry, 2010), consisting of a grid of 60 electrodes, were used in this study to simultaneously record the activity of at least 10–20 dopamine neurons within the acute VTA midbrain slice. The *in vitro* preparation has limitations compared to the intact complete brain. However, the fact that the neurons in the VTA slice are completely devoid of external input has the great advantage that it eliminates background interference from other brain regions. Sufficient dopamine neurons were spontaneously active in the VTA slice to estimate a population measure of functional connectivity, based on the detailed spike timing in the spike trains. The connectivity was quantified with a proven statistical method: the Paired Phase Consistency (Vinck et al., 2010). Two simple manipulations were used that increase the mean firing rate of VTA dopamine neurons to almost the same level: increasing extracellular potassium from 3.5 to 5 mM or bath application of 300 μM glutamate. Although these two manipulations result in a similar change in firing rate, they surprisingly yield distinct modulations of the PPC, confirming the role of functional connectivity as an emerging network property with potential functional consequences for more complex pharmacological manipulations.

## 2. Methods

### 2.1. Slice Preparation

Male wistar rats (Harlan, Zeist, The Netherlands) between 75 and 100 g (age >P24) were decapitated. The midbrain was dissected and kept in artificial cerebral spinal fluid (ACSF) at 4°C, containing (in mM) NaHCO3 25, D-glucose 10, CaCl2 2.5, NaH2PO4 1.25, MgSO4 1.3, KCl 3.5, NaCl 120, which was bubbled with carbogen (95% O 2; 5% CO 2), pH was 7.4. Coronal slices were cut 300 μm thick from caudal to rostral using a vibratome (Leica VT1000S, Wetzlar Germany). The fading of the substantia nigra during progressive slicing was a marker for the caudal-medial part of the VTA. Two to three slices containing the medial to caudal part of the VTA were used for the experiments. Slices were incubated for 30 min at 32°C directly after slicing and were kept at room temperature until the start of the experiment. All experiments and methods were approved by the ethical committee for animal experimentation of the University of Amsterdam.

### 2.2. Solutions

With exception of bicuculline (Tocris Bioscience, Abbington, UK), all chemicals were obtained from Sigma-Aldrich (Zwijndrecht, NL). Stock solutions of quinpirole-HCl (10 mM), glutamate monosodium salt (100 mM) and bicuculline (20 mM) stock were made in H_2_O. All stock solutions were kept at −20° and diluted just before use.

### 2.3. Electrophysiology

During recording (MEA-1600, Multichannel Systems, Reutlingen Germany) the slice was kept at 32°C and continuously perfused with ACSF bubbled with carbogen. The VTA was identified in the midbrain slice and positioned on top of the 3D MEA (Qwane Biosciences, Lausanne, Switzerland) containing 60 electrodes (8*8 layout) of 30 μm diameter and 100 μm spacing in order to record the spontaneous activity of multiple single-units (Olivier et al., 2002). A 20 min acclimatization time preceded the recordings.

### 2.4. Data Acquisition

The extracellular recordings with the 60-channel MEA showed identifiable extracellular spikes of 30 to 130 μV amplitude superimposed on a background noise of about 15 μV. The raw signal was high pass filtered at 225 Hz using a second order Butterworth filter and sampled at 20 kHz. Voltage peaks (positive and negative) were detected, with a relatively low threshold to prevent detection failures. The signal around each peak (± 3 ms) was extracted and K-means clustering was used to cluster the largest two principle components and the maximum amplitudes of the peak waveforms. The auto-correlation and inter-spike-interval distribution of the peaks in the various clusters were examined to identify clusters consisting of neuronal spikes. For electrodes that contained more than one neuron the most reliably recorded neuron was selected, based on the cluster with the largest peak amplitude.

### 2.5. Experimental Conditions

In baseline experiments VTA activity was recorded for 40 min under standard conditions. The selective dopamine D_2_ receptor agonist quinpirole (1 μM) was used to confirm the dopamine sensitivity of the recorded units. It was administered in the last 3 min of the experiment in 40% of the recordings, containing more than 50% of the reported units. Quinpirole induced an unambiguous cessation of action potential firing in all tested slices (*n* = 6) and neurons (*n* = 98). Two manipulations were used to systematically increase the mean firing rate of the DA neurons: (1) in the high potassium experiment [K^+^]_o_ was increased from 3.5 mM (control) to 5 mM and (2) in the glutamate experiments 300 μM glutamate was added to the standard ACSF. Experiments with high extracellular potassium or glutamate consisted of three wash-in and wash-out (25 min each) sequences. The third application was compared to its preceding baseline for the analysis. To determine the potential role of GABA signaling in the observed phenomena, we performed experiments where 20 μM of the GABA antagonist bicuculline was added to the standard ACSF and administered for 20 min. Here the wash-in and wash-out sequence was repeated two times and the second application was compared to its preceding baseline in the analysis.

### 2.6. Data Analysis and Statistics

The firing properties of the VTA neurons were characterized by classical measures: the spike waveform, the mean firing rate (spike/s) and the inter-spike-interval (ISI) distribution. The spike duration was computed by detecting a level crossing in both the beginning and end of the spike waveform. The firing of DA neurons is controlled by an underlying intrinsic rhythm (Drion et al., 2011). The dominant oscillation frequency of this rhythm was estimated from the auto-correlation function (with a 50 ms bin size to accommodate the firing rates in the 1–5 Hz range, **Figure 2A**). The oscillation frequency was computed from intervals between side-lobes in the auto-correlation function (details are given in **Figures 2A,B**). The irregularity of neuronal firing in the VTA was assessed using a measure of local variation, which quantifies the similarity between consecutive ISIs. The local variation (LV, Shinomoto et al., 2005, 2009) of a spike train ranges from 0 (perfectly regular firing) to 1 (Poisson distributed firing) and above 1 for burst-like firing and is given by:

(1) $$\begin{array}{r} LV=\frac{3}{n-1}\overset{n-1}{\underset{i=1}{\sum }}\left(\frac{T_{i}-T_{i+1}}{T_{i}+T_{i+1}}\right) \end{array}$$

where *T*_*i*_ is the i-th interval in the spike train that contains n spikes. Two factors contribute to LV in our situation (1) the local irregularity of the consecutive spikes (jitter) and (2) the consequences of cycle skipping in the firing pattern. The fraction of cycle skipping was calculated as the percentage of the ISIs that were larger than 1.5 times the median ISI. Experimentally, *in vivo*, the local VTA field potential is assumed to best reflect the neuronal Population Output (Fujisawa and Buzsáki, 2011). Our MEA recordings did not provide a field potential of sufficient signal-to-noise ratio, thus we decided to emulate it per slice from all recorded spikes. The Population Output signal of a slice contained the spikes of each recorded neuron in that slice, where a neuron's contribution was normalized to its total number of spikes. This signal was convoluted with a Gaussian kernel (standard deviation 60 ms) to convert it into a continuous signal. The spectral properties of the Population Output signal were computed using Welch's method (Welch, 1967; Hunter, 2007). To determine the strength of the functional connectivity between two neurons that produce a spike train, we used the Paired Phase Consistency (PPC) as previously defined (Vinck et al., 2010). The PPC calculates the similarity of the relative phases of the two trains with respect to a chosen reference frequency and estimates the square of the classic Phase Lock Value (Lachaux et al., 1999). The PPC is an unbiased metric of phase-synchronization that scales with the square rather than the square root of the coherence and phase locking value (Vinck et al., 2010). Thus, a value of 0.0023 corresponds to a coherence value of about 0.048. The PPC indicates the consistency of the relative phase between two spike trains across segments. Assuming a unimodal distribution of relative phases, the probability of having the preferred or most common relative phase for a given segment will be a factor of approximately $\left(1+2*\sqrt{PPC}\right)/\left(1-2*\sqrt{PPC}\right)$ larger than the probability of having the non-preferred or least common relative phase (Ardid et al., 2015). This equation follows from Taylor expansion of the circular von Mises distribution around *PPC* = 0. Hence, with PPC values of 0.0023, the average peak-to-through modulation of the relative phase distribution is approximately 21%. To compute the PPC, spike trains were binned at 1 ms bins and a windowed (Hanning) Fourier Transform was computed on a series of (at least 80) time segments of the spike train. The length of the time segments was set to contain a fixed number of cycles of the reference frequency of interest (e.g., 5 cycles). The relative phase is defined as the complex argument of the classic spectral coherence (Lachaux et al., 1999; Vinck et al., 2010). From these relative phases the PPC was computed:

(2) $$\begin{array}{r} PPC=\frac{2}{N\left(N-1\right)}\overset{N-1}{\underset{j=1}{\sum }}\overset{N}{\underset{k=j+1}{\sum }}cos\left(\theta_{j}-\theta_{k}\right) \end{array}$$

where there are *N* time segments, segment *j* has relative spike phase θ_*j*_ and segment *k* has relative spike phase θ_*k*_, computed in respect to a chosen reference frequency. The PPC was calculated for each unique neuronal pair in the slice and evaluated at three reference frequencies: the oscillation frequency of each neuron in the pair and their mean oscillation frequency. The frequency that yielded the highest PPC value was used for further analysis. The strong auto-correlation of VTA neurons forms a potential bias for the PPC; this was reduced by selecting time segments that were at least 14 s apart. The size of the blind spot (14 s) was based on the time within which the auto-correlation functions of all neurons decayed to less than 15% of its maximum value (**Figures 2A,B**). The statistical significance of an observed PPC value for a neuron pair was investigated by comparing it with the calculation over the shuffled dataset. The bootstrapping for the PPC value included 1,200 computations, where time segments were temporally shuffled each time. The experimentally found PPC value was tested against this shuffled distribution (one-sided, α = 0.05). The relation between LV and PPC of the neurons was investigated using mutual information analysis (MacKay, 2003). The mutual information was computed over all neuron pairs in the baseline experiments (368 pairs) between the LV values of the neurons in a pair (2-dimensional variable) vs. their shared PPC value (one-dimensional variable). As the mutual information can have a strong positive bias (Panzeri et al., 2007), a shuffle correction was applied (Ince et al., 2009). Unless otherwise mentioned, all values reported in this study are given as mean and standard error of the mean (SE).

## 3. Results

### 3.1. Baseline Activity

**Figure 1:**
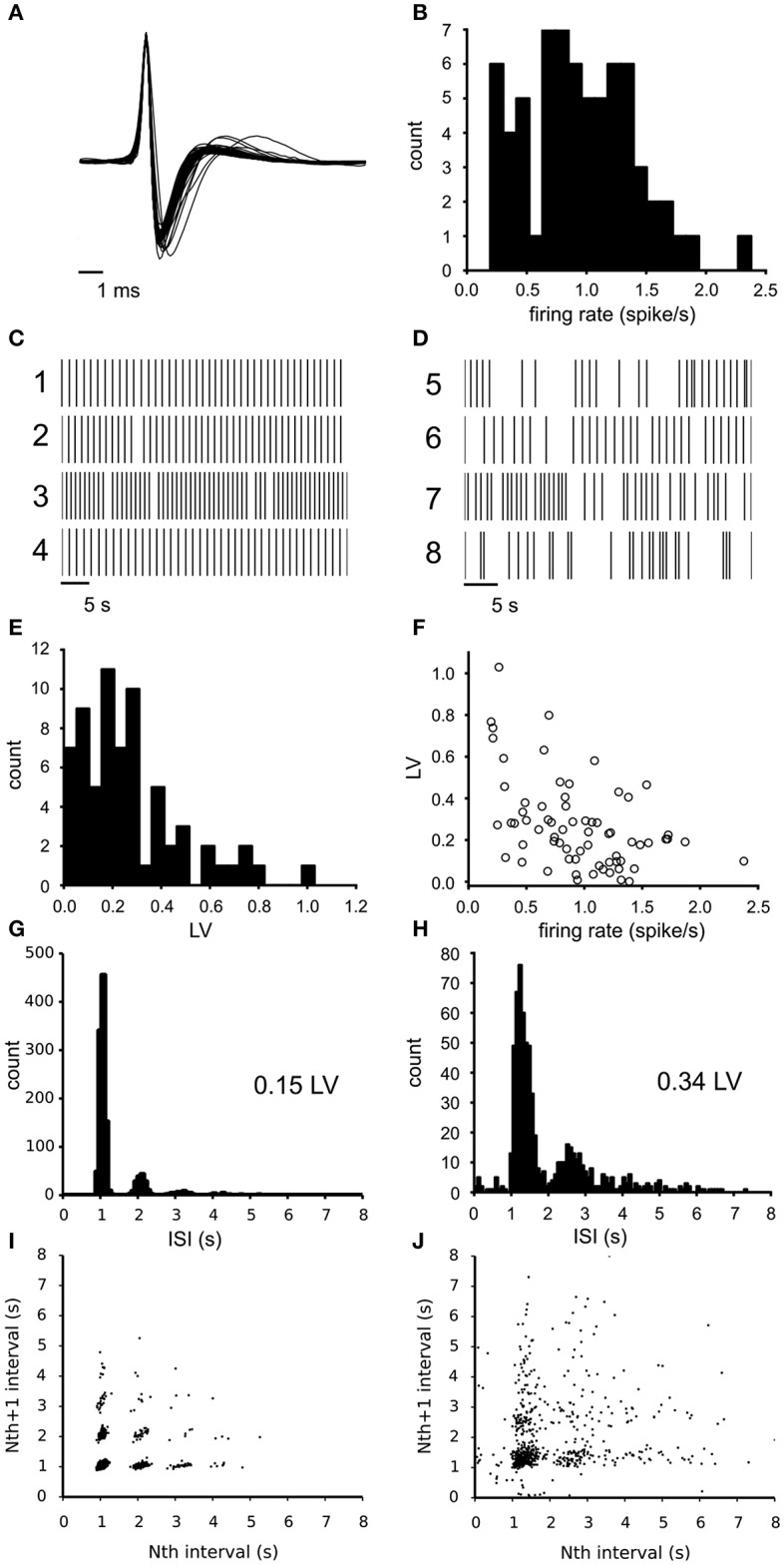
Electrophysiological properties of the recorded VTA dopamine neurons during baseline conditions. **(A)** Superimposed traces of the mean spike waveform per neuron (normalized to the maximum amplitude and centered at the peak). All neurons exhibited the tri-phasic waveform typical of dopamine neurons. **(B)** Distribution of the mean firing rate of all VTA neurons recorded during baseline (*n* = 68). Mean firing rate is computed over a 25–30 min time period. **(C)** Typical firing pattern of four highly regular firing neurons (rasterplot of point processes). **(D)** Typical firing patterns of four irregularly firing neurons (rasterplot of point processes). **(E)** Distribution of LV (firing irregularity) for the same neurons as in **(A)**, determined over the same period. The distribution was not different from unimodal (Hartigan's diptest). **(F)** The relation between firing irregularity (LV) and the mean firing rate showed a negative correlation, indicating that neurons with a lower firing rate fired more irregularly. **(G)** Inter-Spike-Interval (ISI) distribution of a highly regular firing neuron, showing one main peak. **(H)** ISI distribution of a less regular firing neuron (broader main peak), which shows cycle skipping and also includes longer periods of silence. **(I)** return map of a highly regular firing neuron, which relates adjacent spike intervals. A relatively low number of spike cycle skipping (multiples of the preferred interval) was seen in combination with highly discrete point clouds. **(J)** Return map of an irregular firing neuron, which relates adjacent spike intervals. The more diffuse point clouds indicate a less stable oscillation frequency.

The activity of spontaneously active, mesolimbic projecting, dopamine neurons in the lateral VTA was first recorded under baseline conditions (68 neurons from 6 experiments, 8–14 neurons per slice, each from a different animal) using the 60 channel MEA. The dopamine neurons fire action potentials that can extracellularly be recognized by their characteristic broad tri-phasic waveforms and have a spike duration of 2.44 (SE 0.04) ms. Figure 1A illustrates the mean waveform of all 68 neurons superimposed and normalized to their first peak. The classification of all recorded neurons as principal dopamine neurons was confirmed by measuring the quinpirole sensitivity (see methods). The identical waveform of all spikes allows an easy transformation of the recording into a point process, which has been done for all data that follows. The mean firing rate of all observed neurons under baseline condition was 0.93 (SD 0.45) spikes/s (*n* = 68, Figure 1B). Figures 1C,D illustrate two subclasses of firing types that were encountered: (1) quite regularly firing neurons (examples 1–4) and irregularly firing neurons (examples 5–8). For each neuron the irregularity of the firing pattern was quantified by the local variance (Equation 1, LV: mean 0.27 (SD 0.21), *n* = 68), indicating a large variability over the neurons (compare Figures 1C,D). The LV distribution (Figure 1E) did not deviate from a unimodal distribution as tested with the Hartigan's diptest. The firing irregularity correlated negatively with the mean firing rate of the neurons (Spearman'sρ = −0.46, *p* = 6.7^*^10^−5^, Figure 1F), indicating that dopamine neurons with a higher baseline firing rate fired more regularly.

**Figure 2:**
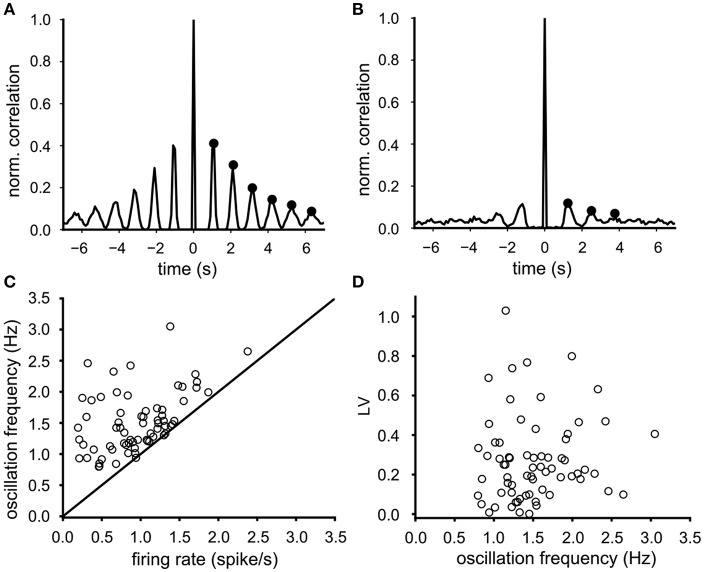
Determination and properties of the oscillation frequency. **(A)** Typical auto-correlation function of a highly regular firing neuron (*LV* = 0.008), where the oscillation frequency (0.9 Hz) was computed from the six side-lobes (indicated by black dots). **(B)** Typical auto-correlation function of a less regular firing neuron (*LV* = 0.34), where the oscillation frequency (0.75 Hz) was computed from three side-lobes (indicated by black dots). **(C)** The oscillation frequency in relation to the mean firing rate for all 68 neurons (baseline condition). The oscillation frequency was either higher or equal to the mean firing rate. This difference was larger for neurons with lower firing rates. **(D)** the firing irregularity (LV) did not correlate with the oscillation frequency.

Figures 1G,H illustrate the inter-spike-interval (ISI) distribution for a regular firing neuron (LV = 0.15) and an irregular firing one (LV = 0.34). At least two factors contribute to the LV value: (1) the width of the dominant ISI peak, which is much narrower for the neuron in Figure 1G than the one in Figure 1H) and (2) the fraction of intervals that are multiples of the median interval and reflect “cycle skipping”. This was corroborated by the correlation found between the LV and the percentage of ISIs exhibiting cycle skipping (Spearman'sρ = 0.82, *p* = 7.6^*^10^−18^). These two factors can also be distinguished in the return maps that were made from the same neurons (Figures 1I,J). The intervals for the neuron in Figures 1G–I were sharply clustered, while that for the neuron in Figures 1H–J were more diffuse. The LV and mean firing rate were not different across slices (ANOVA, α = 0.05, *n* = 6), showing that the observed variation originated at the level of individual neurons.

### 3.2. Neuronal Oscillation Frequency

Most VTA neurons demonstrated a sharp dominant peak in their ISI distribution (e.g., Figures 1G,H) suggesting a preferred spike interval (cycle time), associated with equidistant side lobes in their auto-correlation function. As expected, the side lobes were more prominent in regular firing neurons (Figure 2A, side lobes indicated by dots) than the ones in irregular firing neurons (Figure 2B). The oscillation frequency of the neuronal activity was determined from the time intervals between the side-lobes in the auto-correlation (see markers in Figures 2A,B). The mean oscillation frequency of the recorded neurons was 1.53 (SD 0.47) Hz (*n* = 68) and was either equal but often considerable higher than the mean firing rate (Figure 2C). The difference between the oscillation frequency and the mean firing rate was more pronounced at low firing rates (Figure 2C). In contrast to the mean firing rate, the oscillation frequency did not correlate with the firing irregularity (LV) (Spearman rank regression, *p* = 0.33, Figure 2D), which confirms that the oscillation frequency is less sensitive to cycle skipping than the firing rate and therefore a better and preferred estimator of the intrinsic rhythm of the activity of the VTA dopamine neuron.

### 3.3. Population Output

**Figure 3:**
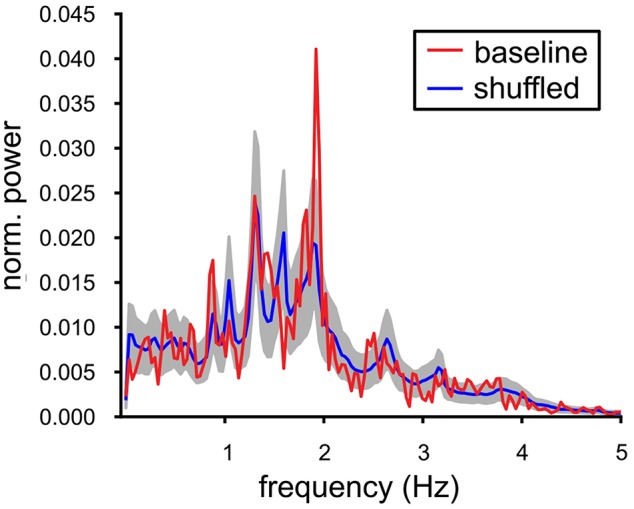
Oscillations in the baseline VTA population output. Power spectrum of the population output of a recorded baseline VTA population (red line, single experiment: 14 neurons). It was contrasted with the power spectrum averaged over 1000 temporally shuffled populations (mean ± SD, resp. blue line and gray filling). The peak in the spectrum of the Population Output at 1.9 Hz was significantly different from the shuffled control and close to the mean oscillation frequency of the VTA neuron population [1.70(SE0.11)] Hz.

*In vivo*, the local field potentials in the VTA contain slow oscillations that are thought to reflect synchronized population activity. Our MEA recordings are not able to provide the equivalent of such a signal and therefore we decided to construct and investigate an alternative Population Output signal based on the joint spike output of all the neurons in the slice. This Population Output signal should be able to indicate signs of underlying neuronal synchrony. The experiment that contained, under baseline conditions, the largest number of spiking VTA neurons (*n* = 14) was analyzed and the power spectrum was computed over a 300 s period. It exhibited a prominent peak oscillation at 1.9 Hz (Figure 3, red line); this frequency was only slightly higher than the mean of the oscillation frequencies calculated for the contributing neurons (1.70 (SE 0.11) Hz). The Population Output spectrum was statistically tested against the spectrum calculated from the shuffled data (Figure 3, blue line, gray band indicates the SD). The shuffling conserved the auto-correlation of the individual neurons, but broke the temporal relationship between the spike trains. The oscillation at 1.9 Hz was the only frequency where the baseline spectrum was significantly different from the shuffled spectrum. The sharply peaked Population Output spectrum suggested an appreciable degree of synchrony between the VTA dopamine neurons.

### 3.4. Baseline Functional Connectivity

**Figure 4:**
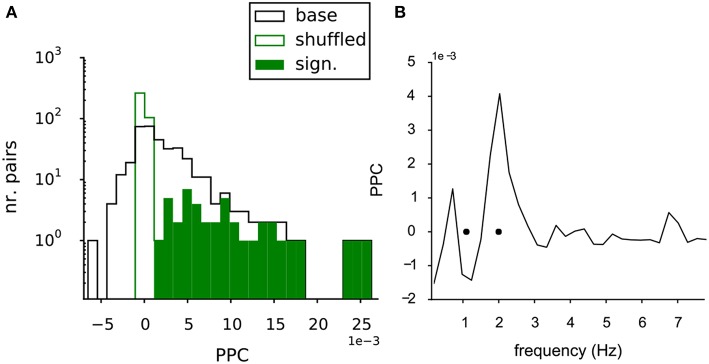
Functional connectivity of the baseline VTA network. **(A)** The PPC values for all pairs within the baseline VTA networks (black line, 368 pairs from 6 experiments) had a mean PPC higher than zero, indicating significant functional coupling. The PPC values of the shuffled controls (green line) were centered at zero, showing the dependency of the phase coupling on the temporal structure in the neuronal spike trains. The significantly bootstrapped pairs (filled green bars, 42 pairs, 11% of total pairs containing 32 out of 68 neurons). **(B)** PPC spectrum for a significantly coupled neuron pair, showing selective coupling (peak in the spectrum) at one of the two oscillation frequencies of the neurons in the pair (black dots).

In the MEA data the potential interaction between the VTA dopamine neurons was further quantified by the functional connectivity between all neuron pairs. The strength of this functional connectivity was measured by their phase coupling, which is based on the relative timing of the spikes in their spike trains. The Paired Phase Consistency (Vinck et al., 2010), was calculated in respect to the oscillation frequencies of the neurons in the pair and over a time period of 1,200-1,500 s. The PPC could be computed for 368 unique pairs of neurons in the recorded population. Figure 4A shows the distribution of the PPC values for the neuron pairs in the baseline condition (Figure 4A, black line). The mean value was larger than zero (2.3 (SE 0.2) *10^−3^; t-test against 0, *p* = 7.2^*^10^−23^). The same statistics were also performed on the shuffled controls (see methods). The PPC values of the shuffled pairs were tightly distributed around zero (green line, Figure 4A), indicating that the measured phase coupling originated from the combined temporal structure in the spike trains.

The statistics of the PPC of an individual pair could also be assessed through bootstrapping: 42 of the 368 (11%) of neuron pairs (involving 32 of the 68 neurons, distributed over all experiments) were significantly coupled (Figure 4A, filled green bars), when directly tested against their shuffled control trains. Figure 4B shows the PPC spectrum for an example neuron pair exhibiting significant coupling. A discrete peak was seen at the oscillation frequency of one of the two neurons (oscillation frequencies indicated by black dots, Figure 4B), indicating that the coupling was selective for the oscillation frequencies of the neurons.

### 3.5. Network Activity Modulation

**Figure 5:**
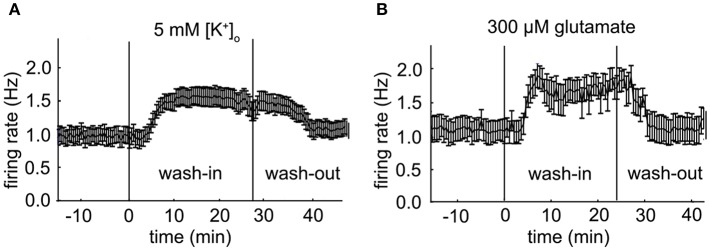
Population firing rate of two VTA dopamine neuron populations. **(A)** Mean firing rate of a VTA dopamine neuron population during an administration of 5 mM extra-cellular potassium [K^+^]_o_ at 30 s time points (error bars indicate SE, baseline: *n* = 20; high potassium: *n* = 22). **(B)** Mean firing rate of a VTA dopamine neuron population during an administration of 300 μM glutamate at 30 s time points (error bars indicate SE, baseline: *n* = 10; glutamate: *n* = 12).

**Figure 6:**
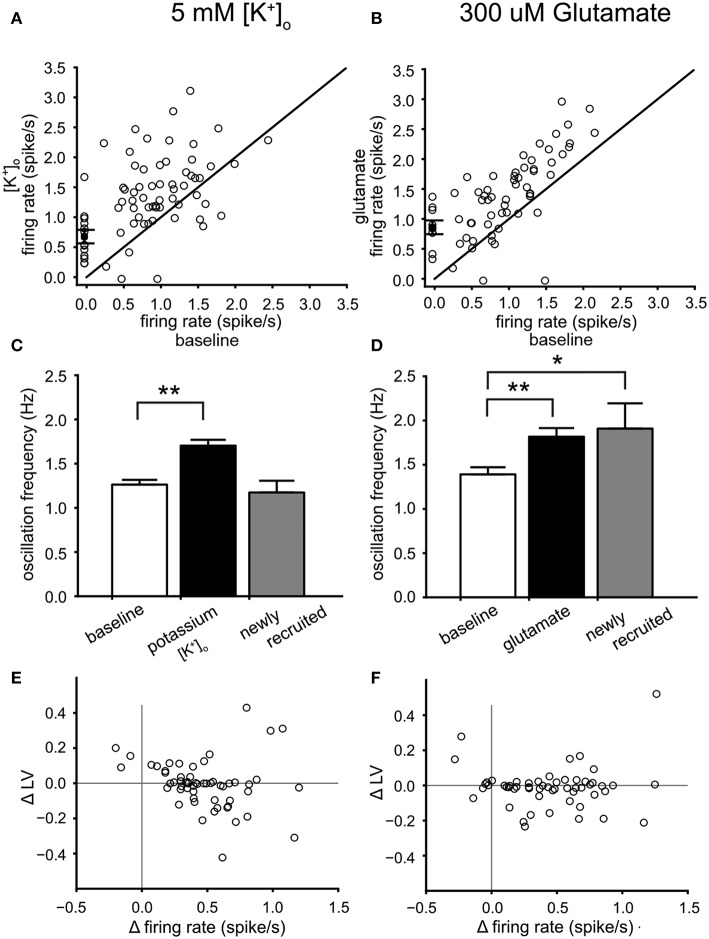
Dopamine neuron activity modulation by high potassium and glutamate. **(A)** Relation between the mean firing rate during 5 mM [K^+^]_o_ and the mean firing rate at baseline. Newly recruited neurons are depicted with a zero mean firing rate at baseline. High potassium induced a 45% increase of the mean firing rate. **(B)** relation between the mean firing rate during 300 μM glutamate and the mean firing rate at baseline. Newly recruited neurons are depicted with a zero mean firing rate at baseline. Glutamate induced a 43% increase of the mean firing rate. **(C)** Comparison of the mean oscillation frequency of VTA neurons at baseline (white bar), with that of the same neurons during 5 mM [K^+^]_o_ (black bar) and the newly recruited neurons by 5 mM [K^+^]_o_ (22% recruited, gray bar). The mean oscillation frequency was increased during 5 mM [K^+^]_o_ for the neurons active during baseline. **(D)** Comparison of the mean oscillation frequency of VTA neurons at baseline (white bar), with that of the same neurons during 300 μM glutamate (black bar) and the newly recruited neurons by 300 μM glutamate (16% recruited, gray bar). Glutamate increased the mean oscillation frequency of the neurons present during baseline. In addition the recruited neurons also had an oscillation frequency higher than baseline. **(E)** The change in firing irregularity (LV) as a function of the change in mean firing rate induced by 5 mM [K^+^]_o_. An increase in mean firing rate correlated with lower firing irregularity. **(F)** The difference (300 μM glutamate minus baseline) in firing irregularity (LV) as a function of the difference in mean firing rate. A correlation was not found. ^*^p < 0.05, ^**^p < 0.01.

The measured PPC can be considered an emerging property of the VTA network. We investigated the relation between the PPC and increased mean neuronal firing rate, using two different forms of excitability modulation. First, raising [K^+^]_o_ from a baseline level of 3.5 to 5 mM, increased the firing rate (4 experiments, 57 neurons under baseline condition). Second, bath application of 300 μM glutamate increases firing rate to about the same level (5 experiments, 55 neurons under baseline condition). The enhanced excitability induced by both procedures was reversible, although some of the newly recruited neurons did not silence at wash-out. The time course of the mean firing rate of a VTA dopamine neuron population can be observed during a high potassium (Figure 5A) and a glutamate application (Figure 5B). Enhancing the potassium concentration to 5 mM, increased the mean firing rate of the VTA neurons by about 45% (Figure 6A) and the oscillation frequency by about 33% (Figure 6C) (statistics: ANOVA, *n* = 57, firing rate: *p* = 3.4*10^−6^ oscillation frequency: *p* = 1^*^10^−6^). Application of 300 μM glutamate induced similar changes in firing rate (Figure 6B) and oscillation frequency (Figure 6D) (statistics: ANOVA, *n* = 55, firing rate: *p* = 5.8^*^10^−5^, oscillation frequency: *p* = 0.001). In addition to increasing the neuronal activity, both treatments recruited 22% (high potassium) and 16% (glutamate) additional neurons, shown in Figures 6A,B as neurons with a baseline firing rate set to zero. The neurons recruited by glutamate had an oscillation frequency higher than baseline [Figure 6D; ANOVA, *p* = 0.027, *n* = (55 baseline, 9 recruited)], whereas those recruited by high potassium had an oscillation frequency similar to baseline [Figure 6C, *n* = (57 baseline, 13 recruited)]. The increase in firing rate due to high potassium correlated with a decrease in LV (Spearman'sρ = −0.35, *p* = 0.0074, *n* = 57, Figure 6E), but this relationship was not present for the increase in firing rate induced by glutamate (Spearman'sρ = −0.05, *p* = 0.71, *n* = 55, Figure 6F). The two correlation coefficients were different (α = 0.05, Fisher transform). These results indicated that, although the effect on the firing rate was quite similar for high potassium and glutamate, the manipulations had a differential effect on firing irregularity.

### 3.6. Population Output Modulation

**Figure 7:**
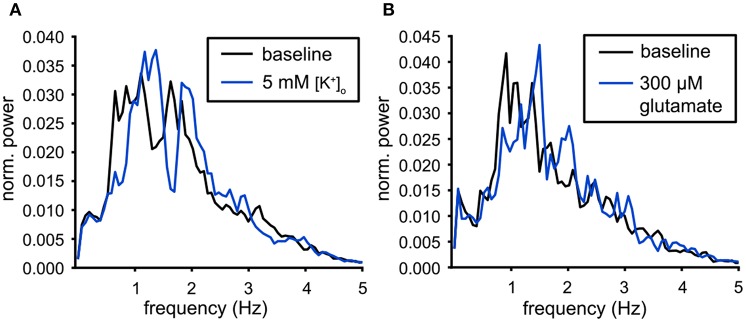
Modulation of the population output. **(A)** Power spectra of the population output for baseline conditions (black) and for high potassium (5 mM [K^+^]_o_, blue). The dominant peak was shifted to a higher frequency during the administration of 5 mM [K^+^]_o_ (baseline: *n* = 19, 5 mM [K^+^]_o_ : *n* = 25). **(B)** Power spectra of the population output for baseline conditions (black) and for 300 μM glutamate (blue). The dominant peak was shifted to a higher frequency during the administration of glutamate (baseline: *n* = 22, glutamate: *n* = 26).

The population output of the VTA neuronal population was compared under the two excitatory conditions. The analysis was performed on the slices with the largest number of neurons (19 neurons for high [K^+^]_o_ and 25 neurons for the 300 μM glutamate condition, active during the preceding baseline condition). The normalized power spectra were computed from the Population Output over a 20 min time period. Figure 7 illustrates that the dominant peak in the spectrum shifts to a higher frequency for both the high potassium (Figure 7A) and glutamate condition (Figure 7B). These shifts confirmed the changes in mean oscillation frequency of the individual neurons (Figures 6C,D). The normalized Population Output spectra allowed to compare the shape of the dominant peak, but in order to investigate the consequences of increased excitability for the functional connectivity, we determined the pairwise functional connectivity.

### 3.7. Connectivity Modulation

**Figure 8:**
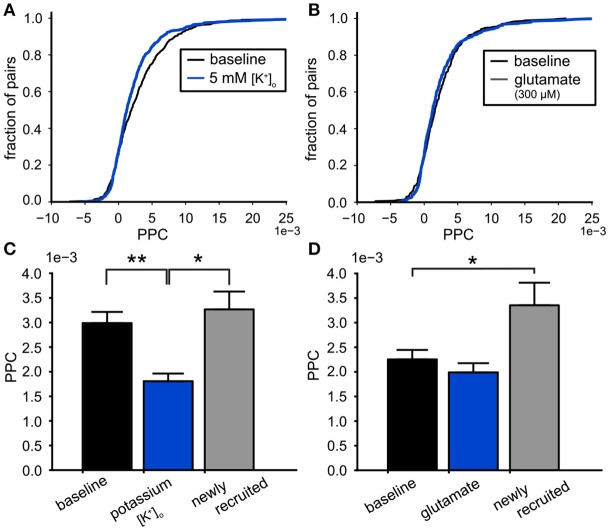
Effects on functional connectivity induced by high potassium and glutamate. **(A)** The cumulative PPC for the high potassium (5 mM [K^+^]_o_ ) and baseline conditions. The leftward shift of the distribution shows that the pairwise connectivity was weakened during the high potassium condition (baseline: 442 pairs, high potassium: 652 pairs). **(B)** The cumulative PPC histograms for the baseline and 300 μM glutamate conditions. The two distributions were identical, indicating that the network strength was similar for both conditions (baseline: 367 pairs, glutamate: 486 pairs). **(C)** Comparison of the mean PPC for neuron pairs during baseline conditions (black bar, *n* = 415), with the same pairs during 5 mM [K^+^]_o_ (blue bar, *n* = 415) and the connections made by neurons newly recruited by 5 mM [K^+^]_o_ (gray bar, *n* = 237). The weakening of the functional connectivity by high potassium originated from the functional connections already present during baseline. **(D)** Comparison of the mean PPC for neuron pairs during baseline conditions (black bar, *n* = 349), with the same pairs during 300 μM glutamate administration (blue bar, *n* = 349) and the connections made by neurons newly recruited by 300 μM glutamate (gray bar, *n* = 137). The functional connections already present during baseline were not affected by glutamate (black), however the connections made by the recruited neurons were stronger than baseline (gray). ^*^p < 0.05, ^**^p < 0.01.

The modulation of functional connectivity in the VTA network by high potassium or glutamate was analyzed by computing the PPC values of all pairs of neurons within the VTA populations for the conditions of increased excitability and their preceding baseline. The PPC was calculated over 1,200–1,500 s windows for the baseline as well as the excitatory condition. The cumulative PPC distribution for all neuron pairs in the high potassium condition compared to baseline, shows the shift to lower PPC values during high potassium (baseline PPC: 3.0 (SE 0.2) ^*^10^−3^, 442 pairs, 5 mM [K^+^]_o_ PPC: 2.3 (SE 0.2) ^*^10^−3^, 652 pairs, Kolmogorov-Smirnov, *p* = 5.3^*^10^−4^, Figure 8A). Comparing the PPC distribution before and during glutamate application confirmed the absence of an effect on the PPC (baseline PPC: 2.4 (SE 0.2) ^*^10^−3^, 367 pairs, with glutamate PPC:2.3(*SE*0.2)^*^10^−3^, 486 pairs, Kolmogorov-Smirnov, *p* = 0.24, Figure 8B). To assess the differential effect of glutamate and potassium on the PPC we performed multi-level analyses on neuron pairs active before and after the treatment (Field et al., 2012). First, we performed the analysis on the datasets of potassium and glutamate separately. PPC values of the neuron pairs, before and after treatment represented a repeated measure. The test included a fixed effect for the treatment and a random effect for the baseline PPC (across slices, potassium: 4 slices, glutamate: 5 slices). The latter controls for potential differences in baseline PPC values between slices. High potassium reduced the PPC of neuron pairs (δPPC:−1.2(*SE*0.3)^*^10^−3^, *n* = 415, *p* < 0.01). In contrast, glutamate treatment did not significantly affect the PPC within neuron pairs (δPPC:−0.26(*SE*0.3)^*^10^−3^, *n* = 349, *p* = 0.28). Adding a fixed effect for the substance type (potassium and glutamate) allowed us to determine the interaction between the type of substance and treatment (repeated measure). The multi-level analysis demonstrated a significant interaction (*p* = 0.037) between the substance type and treatment (repeated measure), substantiating the difference between potassium and glutamate. Similar results were obtained when we repeated the analysis controlling for random effects of PPC baseline values of slices and pairs. Both conditions recruited neurons due to their excitatory effect (high [K^+^]_o_ : 22% and glutamate: 16%). These recruited neurons were engaged in new functional connections with each other and with the neurons already active under baseline conditions. Figures 8C,D contain bar plots of the PPC divided up for the pairs of neurons that were present in both the baseline and experimental conditions and the new pairs made with the recruited neurons. This division showed that the lower PPC value during high potassium was due to a reduction in PPC of the baseline network and not due to newly recruited neurons (ANOVA, *p* = 2.3^*^10^−5^, *n* = 415, Figure 8C). The neurons recruited by glutamate generated higher PPC values compared to baseline (ANOVA, *p* = 0.01, *n* = 349, baseline, *n* = 137, Figure 8D). High potassium decreased the LV of the neurons active under baseline conditions (Figure 6E). Additionally, it reduced the PPC between these neurons (Figure 8C). We investigated the possible relation between the firing irregularity and PPC through mutual information analysis. The mutual information measured the strength of the association between the LV values of the neurons in a pair and their PPC value. It was computed for all neuron pairs during the baseline condition (*n* = 368). The LV values predicted 2.8 out of the 8.4 bits of total entropy in the PPC distribution after mutual information shuffle correction (Panzeri et al., 2007).

### 3.8. GABA-A Modulation

**Figure 9:**
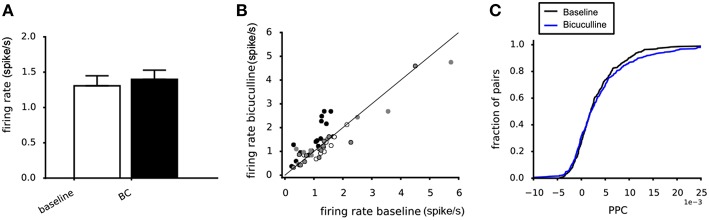
Effect of Bicuculline on the dopamine neuron population activity and connectivity. **(A)** The mean firing rate was not affected by 20 μM Bicuculline, when averaging across four experiments (white vs. black bar). **(B)** Relation between the mean firing rate during 20 μM Bicuculline and the mean firing rate at baseline for four experiments (filled and non-filled dots). A systematic response to Bicuculline was not found. **(C)** The cumulative PPC histograms for the baseline and 20 μM bicuculline conditions. The two distributions were not different, indicating that bicuculline had no effect on the network strength when averaged over four experiments (baseline: 305 pairs, bicuculline: 305 pairs).

Around 20–30% of the neurons in the VTA are known to be GABAergic interneurons (Nair-Roberts et al., 2008). They could play a role in explaining the difference between the potassium and glutamate experiments. To assess their contribution to the observed effects on the PPC we used the GABA-A antagonist bicuculline. Bicuculline administration (20 μM) did not affect the mean firing rates of the dopamine neurons, averaged across four experiments (Figure 9A). The scatter plot confirms the absence of systematic changes of the neuronal firing rate (Figure 9B). Bicuculline (20 μM) also did not affect the PPC, when averaged over four experiments (Kolmogorov-Smirnov, *p* = 0.72, Figure 9C).

## 4. Discussion

In this study we quantify the PPC between pairs of neurons and interpret it as a measure of functional connectivity; it appears as an emerging property during excitability modulation of dopaminergic neurons in acute brain slices of the VTA. Two manipulations that induced a similar increase of the mean neuronal firing rate, had a differential effect on the PPC. This could make the organization of network synchrony as captured in the PPC a unique element of the population message that is send to the projection areas.

VTA dopamine neurons possess a strong intrinsic rhythm generator, illustrated by the spontaneous oscillating firing patterns in the isolated slice. These rhythms even persist in dissociated single neurons (Koyama et al., 2005). It has been hypothesized that the VTA circuit could generate a low frequency rhythm important for memory, which entrains the prefrontal cortex and the hippocampus (Fujisawa and Buzsáki, 2011). The functional connectivity local to the VTA that we find can be an important factor in generating such a rhythm. The mean firing rate of all dopaminergic neurons recorded in our preparation (~1 Hz) is lower than the one reported *in vivo*: 5 (SD 6) Hz (Fujisawa and Buzsáki, 2011). The same holds for the mean oscillation frequency (~2 Hz in our experiments) vs. the oscillations in the field potential reported *in vivo*: 4 Hz. We carefully restricted our recordings to the “classical” lateral part of the VTA that contains an uniform class of mesolimbic projection neurons (Lammel et al., 2008). All recorded neurons were identified as principal neurons based on their electrophysiological properties: characteristic broad spike waveform and regular low frequency firing pattern. In addition we could unambiguously link these properties to a positive response to the D_2_-receptor agonist quinpirole in all tested neurons. Although the accuracy of these markers has been debated, our findings correspond to the abundance (~60%) of these neurons in the VTA (Margolis et al., 2006). The VTA also contains glutamatergic and GABAergic neurons that can make up 20 to 30% of the population (Nair-Roberts et al., 2008). They are typically faster spiking (> 10 Hz) neurons and we never picked up more than one such unit per slice; too few to incorporate them in a statistical sound way into this study. The acute brain slice is very suitable for work with the Micro Electrode Array, but has limitations. In the slicing process we may have lost quite a few connections and the preparation is devoid of its normal background input. The first aspect will lead to an underestimation of the functional connectivity, while the second one brings a careful analysis within reach. The MEA has only two dimensional electrodes with a diameter of 30 μM and spacing of 100 μm, which inevitably leads to sparse sampling of the population; nevertheless we can simultaneously record ensembles of 10–30 spiking dopamine neurons.

Most of our VTA dopaminergic neurons had a distinct firing rate and LV, but these values varied considerably over the population, even within the same slice. Our distribution of firing rate and LV was, however, unimodal in contrast to the observations made in acutely dissociated neurons, where two distinct populations have been described (Koyama et al., 2005). The assumption of an underlying oscillatory process that triggers the action potentials, predicts that the auto-correlation of the spike train is a better estimator of the oscillation frequency than the firing rate, which we confirmed. The LV is determined by local irregularity of consecutive spikes and by cycle skipping. This is in accordance with the finding that a lower baseline firing rate correlated with a higher firing irregularity, but that this correlation did not exist between the oscillation frequency and the irregularity. Rhythmic action potential firing in VTA neurons originates from a balance between an inward sodium current and a calcium-based slow oscillatory potential (SOP) (Drion et al., 2011). There is evidence that sodium currents form the dominant mechanism and generate quite regular spike firing in the VTA (Khaliq and Bean, 2010; Drion et al., 2011), while in dopamine neurons from the substantia nigra spikes are generated predominantly by the less regular SOP (Putzier et al., 2009; Drion et al., 2011).

We measured the strength of the synchrony between all possible pairs of VTA neurons in the same slice with the Paired Phase Consistency (PPC) (Lachaux et al., 1999; Vinck et al., 2010). The mean value of the PPC of the baseline VTA network was different from zero which indicates the presence of significant functional coupling in the network. When tested individually, 42 out of 382 all possible pairs demonstrated significant coupling at their oscillation frequency (~2 Hz). Of the 68 neurons sampled in all experiments, 32 were part of a pair with significant coupling, which shows that the VTA contains a collection of functionally connected dopamine neurons that synchronizes at the time scale of their preferred oscillation frequency. The details of the underlying mechanism of this coupling cannot be deduced from the current measurements alone. Direct synaptic connectivity as demonstrated for dopaminergic neurons in the Substantia Nigra (Vandecasteele et al., 2008) could be involved as could be electrical coupling through gap junctions. However in a previous study (van der Velden et al., 2017) we concluded that probably the best explation of the apparent synchrony in the VTA network is through a mechanism that is called dopamine volume transmission (Zoli et al., 1998). In volume transmission the collective and synchronized release of dopamine in the extracellular space creates an oscillating dopamine concentration, where the precize role of each neuron depends on its cellular sensitivity for dopamine. In the current paper we refer to our form of coupling as functional connectivity in contrast to the more straigtforward anatomical/synaptic connectivity.

Most pharmacological studies of the VTA have focused on describing modulations of individual neuronal firing rates (Hand et al., 1987; Wang and French, 1993; Werkman et al., 2001). The MEA recordings allow us to incorporate functional connectivity into this modulation. Here we used two well understood manipulations of neuronal activity: increasing extracellular potassium from 3.5 mM to 5 mM and bath-application of 300 μM glutamate, to study the relation between neuronal activity and functional connectivity. The manipulations were chosen so that their effect on mean firing rate and mean oscillation frequency was similar. Interestingly, they had a differential effect on firing irregularity (LV) as well as on functional connectivity as measured with the PPC. The increase in firing rate induced by high potassium correlated with a lower LV, while such correlation was absent in the case of glutamate application. The increased activity of individual neurons propagates through the population level, as high potassium and glutamate both increased the oscillation frequency of the population output. Calculating the connectivity strength using the PPC demonstrated that the functional connectivity was weakened by high potassium, while it was unaffected by glutamate. The weakening of the functional connectivity originated from neurons already present during the baseline condition and not from connections made by neurons recruited by high potassium. Depolarization of the membrane potential by high potassium can strengthen the role of the ‘persistent’ sodium current in the generation of spikes (Khaliq and Bean, 2010) and this can explain the reduction in firing irregularity (Drion et al., 2011). Glutamate depolarizes the neuron through the activation of post-synaptic AMPA and NMDA receptors (Wang and French, 1993). The strongly increased synaptic activity enhances firing irregularity (Drion et al., 2011) and could therefore explain the difference between the effect of glutamate- and potassium-induced depolarization. The strengthening of the intrinsic rhythm by larger sodium currents could make the dopamine neurons less sensitive to synchronizing inputs and thus show up as weaker neuronal interactions, based on resonance principles (Hunter et al., 1998; Coombes and Bressloff, 1999). Neurons recruited by glutamate had a higher than baseline oscillation frequency and functional connectivity, suggesting that recruitment through increased synaptic input (glutamate) leads to more network participation than recruitment through a direct increase of the membrane potential (high potassium).

GABAergic transmission contributed on average very little to the functional connectivity. Our activity modulation demonstrates that physiological relevant stimuli (high potassium and glutamate) can alter the functional connectivity of the local VTA network. This effect seems independent from the modulation of the neuronal firing rate. The difference between the effects on the functional connectivity by high potassium and glutamate, indicates that the specific mechanisms with which these substances excite the individual dopamine neurons (electro-chemical vs. synaptic) is of importance for the response at the network level. Functional connectivity shows up as an aspect of spatial synchronization that is present in the output of the VTA and can thus form a unique element of the message that is sent to the projection areas. Functional connectivity between VTA dopamine neurons is involved in reward processing (Kim et al., 2012; Moghaddam et al., 2017) and we analyze this connectivity in detail. Functional connectivity adds a dimension to pharmacological manipulation of the VTA micro circuit and could lead to a better understanding of pharmacological (side) effects of e.g., anti-psychotic drugs on the mesolimbic and mesocortical projection areas.

## Data Availability

The datasets generated for this study are available on request to the corresponding author.

## Ethics Statement

All experiments and methods were approved by the ethical committee for animal experimentation of the University of Amsterdam.

## Author Contributions

LvdV, MV, TW, and WW: designed the study. LvdV, MV, and WW: contributed to the data analysis and writing of the manuscript. LvdV: performed the experiments and the data analysis.

### Conflict of Interest Statement

The authors declare that the research was conducted in the absence of any commercial or financial relationships that could be construed as a potential conflict of interest.

## References

[B1] Ardid S. Vinck M. Kaping D. Marquez S. Everling S. Womelsdorf T. (2015). Mapping of functionally characterized cell classes onto canonical circuit operations in primate prefrontal cortex. J. Neurosci. 35, 2975–2991. 10.1523/JNEUROSCI.2700-14.2015 25698735 PMC6605590

[B2] Bayer H. M. Lau B. Glimcher P. W. (2007). Statistics of midbrain dopamine neuron spike trains in the awake primate. J. Neurophysiol. 98, 1428–1439. 10.1152/jn.01140.2006 17615124

[B3] Bayer V. E. Pickel V. M. (1990). Ultrastructural localization of tyrosine hydroxylase in the rat ventral tegmental area: relationship between immunolabeling density and neuronal associations. J. Neurosci. 10, 2996–3013. 10.1523/JNEUROSCI.10-09-02996.1990 1975839 PMC6570237

[B4] Björklund A. Dunnett S. B. (2007). Dopamine neuron systems in the brain: an update. Trends Neurosci. 30, 194–202. 10.1016/j.tins.2007.03.006 17408759

[B5] Bowery B. Rothwell L. a. Seabrook G. R. (1994). Comparison between the pharmacology of dopamine receptors mediating the inhibition of cell firing in rat brain slices through the *Substantia nigra* pars compacta and ventral tegmental area. Brit. J. Pharmacol. 112, 873–880. 10.1111/j.1476-5381.1994.tb13161.x 7921615 PMC1910205

[B6] Coombes S. Bressloff P. C. (1999). Mode locking and Arnold tongues in integrate-and-fire neural oscillators. Phys. Rev. E 60(2 Pt B):2086–2096. 10.1103/PhysRevE.60.2086 11970001

[B7] Drion G. Massotte L. Sepulchre R. Seutin V. (2011). How modeling can reconcile apparently discrepant experimental results: the case of pacemaking in dopaminergic neurons. PLoS Comput. Biol. 7:e1002050. 10.1371/journal.pcbi.1002050 21637742 PMC3102759

[B8] Field A. Miles J. Field Z. (2012). Discovering Statistics Using R. Thousand Oaks, CA: SAGE Publications.

[B9] Fields H. L. Hjelmstad G. O. Margolis E. B. Nicola S. M. (2007). Ventral tegmental area neurons in learned appetitive behavior and positive reinforcement. Annu. Rev. Neurosci. 30, 289–316. 10.1146/annurev.neuro.30.051606.094341 17376009

[B10] Fujisawa S. Buzsáki G. (2011). A 4 Hz oscillation adaptively synchronizes prefrontal, VTA, and hippocampal activities. Neuron 72, 153–165. 10.1016/j.neuron.2011.08.018 21982376 PMC3235795

[B11] Gonon F. (1988). Nonlinear relationship between impulse flow and dopamine released by rat midbrain dopaminergic neurons as studied by *in vivo* electrochemistry. Neuroscience 24, 19–28. 10.1016/0306-4522(88)90307-7 3368048

[B12] Grace A. A. Onn S. p. (1989). Morphology and electrophysiological properties of lmmunocytochemically identified rat dopamine neurons recorded in wifro. J. Neurosci. 9, 3463–3481. 10.1523/JNEUROSCI.09-10-03463.1989 2795134 PMC6569889

[B13] Hand T. H. Hu X. T. Wang R. Y. (1987). Differential effects of acute clozapine and haloperidol on the activity of ventral tegmental (A10) and nigrostriatal (A9) dopamine neurons. Brain Res. 415, 257–269. 10.1016/0006-8993(87)90207-1 3607497

[B14] Hunter J. D. (2007). Matplotlib: A 2D graphics environment. Comput. Sci. Eng. 9, 90–95. 10.1109/MCSE.2007.55

[B15] Hunter J. D. Milton J. G. Thomas P. J. Cowan J. D. (1998). Resonance effect for neural spike time reliability. J. Neurophysiol. 80, 1427–1438. 10.1152/jn.1998.80.3.1427 9744950

[B16] Ince R. A. A. Petersen R. S. Swan D. C. Panzeri S. (2009). Python for information theoretic analysis of neural data. Front. Neuroinformatics 3:4. 10.3389/neuro.11.004.2009 19242557 PMC2647335

[B17] Khaliq Z. M. Bean B. P. (2010). Pacemaking in dopaminergic ventral tegmental area neurons: depolarizing drive from background and voltage-dependent sodium conductances. J. Neurosci. 30, 7401–7413. 10.1523/JNEUROSCI.0143-10.2010 20505107 PMC2892804

[B18] Kim Y. Wood J. Moghaddam B. (2012). Coordinated activity of ventral tegmental neurons adapts to appetitive and aversive learning. PloS ONE 7:e29766. 10.1371/journal.pone.0029766 22238652 PMC3253108

[B19] Koyama S. Kanemitsu Y. Weight F. F. (2005). Spontaneous activity and properties of two types of principal neurons from the ventral tegmental area of rat. J. Neurophysiol. 93, 3282–3293. 10.1152/jn.00776.2004 15659533

[B20] Lachaux J. P. Rodriguez E. Martinerie J. Varela F. J. (1999). Measuring phase synchrony in brain signals. Hum. Brain Mapp. 8, 194–208. 10619414 10.1002/(SICI)1097-0193(1999)8:4<194::AID-HBM4>3.0.CO;2-C PMC6873296

[B21] Lammel S. Hetzel A. Häckel O. Jones I. Liss B. Roeper J. (2008). Unique properties of mesoprefrontal neurons within a dual mesocorticolimbic dopamine system. Neuron 57, 760–773. 10.1016/j.neuron.2008.01.022 18341995

[B22] Lisman J. E. Grace A. A. (2005). The hippocampal-VTA loop: controlling the entry of information into long-term memory. Neuron 46, 703–713. 10.1016/j.neuron.2005.05.002 15924857

[B23] MacKay D. J. C. (2003). Information Theory, Inference and Learning Algorithms. Cambridge, UK; New York, NY: Cambridge University Press.

[B24] Margolis E. B. Lock H. Hjelmstad G. O. Fields H. L. (2006). The ventral tegmental area revisited: is there an electrophysiological marker for dopaminergic neurons? J. Physiol. 577(Pt 3):907–924. 10.1113/jphysiol.2006.117069 16959856 PMC1890372

[B25] Moghaddam B. Koerner F. S. Kass R. E. Wood J. Simon N. W. (2017). Networks of VTA neurons encode real-time information about uncertain numbers of actions executed to earn a reward. Front. Behav. Neurosci. 11, 1–16. 10.3389/fnbeh.2017.00140 28848408 PMC5550723

[B26] Nair-Roberts R. G. Chatelain-Badie S. D. Benson E. White-Cooper H. Bolam J. P. Ungless M. A.. (2008). Stereological estimates of dopaminergic, GABAergic and glutamatergic neurons in the ventral tegmental area, substantia nigra and retrorubral field in the rat. Neuroscience 152, 1024–1031. 10.1016/j.neuroscience.2008.01.046 18355970 PMC2575227

[B27] Olivier M. Fejtl M. Raggenbass M. Bertrand D. Renaud P. Heuschkel M. O. (2002). A three-dimensional multi-electrode array for multi-site stimulation and recording in acute brain slices. J. Neurosci. Methods 114, 135–148. 10.1016/S0165-0270(01)00514-3 11856564

[B28] Omelchenko N. Sesack S. R. (2009). Ultrastructural analysis of local collaterals of rat ventral tegmental area neurons: GABA phenotype and synapses onto dopamine and GABA cells. Synapse 63, 895–906. 10.1002/syn.20668 19582784 PMC2741309

[B29] Paladini C. A. Roeper J. (2014). Generating bursts (and pauses) in the dopamine midbrain neurons. Neuroscience 282C, 109–121. 10.1016/j.neuroscience.2014.07.032 25073045

[B30] Panzeri S. Senatore R. Montemurro M. A. Petersen R. S. (2007). Correcting for the sampling bias problem in spike train information measures. J. Neurophysiol. 98, 1064–1072. 10.1152/jn.00559.2007 17615128

[B31] Pucak M. L. Grace A. A. (1994). Evidence that systemically administered dopamine antagonists activate dopamine neuron firing primarily blockade of somatodendritic. J. Pharmacol. Exp. Therapeut. 271, 1181–1192. 7996424

[B32] Pucak M. L. Grace A. a. (1996). Effects of haloperidol on the activity and membrane physiology of substantia nigra dopamine neurons recorded *in vitro*. Brain Res. 713, 44–52. 10.1016/0006-8993(95)01460-8 8724974

[B33] Putzier I. Kullmann P. H. M. Horn J. P. Levitan E. S. (2009). Cav1.3 channel voltage dependence, not Ca2+ selectivity, drives pacemaker activity and amplifies bursts in nigral dopamine neurons. J. Neurosci. 29, 15414–15419. 10.1523/JNEUROSCI.4742-09.2009 20007466 PMC2796195

[B34] Shinomoto S. Kim H. Shimokawa T. Matsuno N. Funahashi S. Shima K.. (2009). Relating neuronal firing patterns to functional differentiation of cerebral cortex. PLoS Comput. Biol. 5:e1000433. 10.1371/journal.pcbi.1000433 19593378 PMC2701610

[B35] Shinomoto S. Miura K. Koyama S. (2005). A measure of local variation of inter-spike intervals. Bio Systems 79, 67–72. 10.1016/j.biosystems.2004.09.023 15649590

[B36] Taketani M. Baudry M. (2010). Advances in Network Electrophysiology. New York, NY: Springer.

[B37] Traub R. D. Miles R. Wong R. K. (1989). Model of the origin of rhythmic population oscillations in the hippocampal slice. Science 243, 1319–1325. 10.1126/science.2646715 2646715

[B38] van der Velden L. Vinck M. Werkman T. R. Wadman W. J. (2017). Tuning of neuronal interactions in the lateral ventral tegmental area by dopamine sensitivity. Neuroscience 366, 62–69. 10.1016/j.neuroscience.2017.10.009 29037597

[B39] Vandecasteele M. Glowinski J. Deniau J. M. Venance L. (2008). Chemical transmission between dopaminergic neuron pairs. Proc. Natl. Acad. Sci. U.S.A. 105, 4904–4909. 10.1073/pnas.0703121105 18347345 PMC2290772

[B40] Vinck M. van Wingerden M. Womelsdorf T. Fries P. Pennartz C. M. A. (2010). The pairwise phase consistency: a bias-free measure of rhythmic neuronal synchronization. NeuroImage 51, 112–122. 10.1016/j.neuroimage.2010.01.073 20114076

[B41] Wang T. French E. D. (1993). L-glutamate excitation of A10 dopamine neurons is preferentially mediated by activation of NMDA receptors: extra- and intracellular electrophysiological studies in brain slices. Brain Res. 627, 299–306. 10.1016/0006-8993(93)90334-J 7905352

[B42] Welch P. D. (1967). The use of fast fourier transform for the estimation of power spectra: a method based on time averaging over short, modified periodograms. IEEE Trans. Audio Electroacoust. 15, 70–73. 10.1109/TAU.1967.1161901

[B43] Werkman T. R. Kruse C. G. Nievelstein H. Long S. K. Wadman W. J. (2001). *In vitro* modulation of the firing rate of dopamine neurons in the rat substantia nigra pars compacta and the ventral tegmental area by antipsychotic drugs. Neuropharmacology 40, 927–936. 10.1016/S0028-3908(01)00015-6 11378163

[B44] Zoli M. Torri C. Ferrari R. Jansson A. Zini I. Fuxe K.. (1998). The emergence of the volume transmission concept. Brain Res. Rev. 26, 136–147. 10.1016/S0165-0173(97)00048-9 9651506

